# Primary extraskeletal Ewing's sarcoma of the breast in a 13-year-old girl: a case report

**DOI:** 10.3389/fped.2025.1499612

**Published:** 2025-08-29

**Authors:** Xiaoge Liu, Xin Li, Chaoxin Zhou, Dan Liu

**Affiliations:** ^1^ Department of Ultrasound, Ya'an People’s Hospital, Ya'an, Sichuan, China; ^2^ Department of Radiology, Sichuan Provincial People’s Hospital, University of Electronic Science and Technology, Chengdu, Sichuan, China; ^3^ Department of Radiology, The First People’s Hospital of Liangshan Yi Autonomous Prefecture, Xichang, Sichuan, China

**Keywords:** extraskeletal Ewing sarcoma, primitive neuroectodermal tumor, pediatric, breast cancer, surgery, MRI

## Abstract

**Background:**

The Ewing sarcoma family of tumors (ESFT) comprises classic Ewing sarcoma (ES) of the bone and extraskeletal Ewing sarcoma (EES). EES typically arises in the soft tissues of the trunk and extremities. Primary breast ES is a rare entity, predominantly reported as clinical case reports. Furthermore, pediatric primary breast ES is exceptionally rare. To date, there have been few reports of clinical cases.

**Case presentation:**

we report a rare case of primary breast ES in a 13-year-old girl from a Chinese ethnic minority group.She presented with an accidentally discovered enlarging mass in her right breast. Initial evaluations at a local hospital, including breast ultrasound and chest CT scan, revealed an 11.8 × 10.3 × 8.5 cm solid and cystic mass within the right breast.This was initially misdiagnosed as a fibroadenoma or a phyllodes tumor, likely due to her younger age. Laboratory findings showed elevated levels of lactate dehydrogenase (LDH) and alkaline phosphatase (ALP). No other extra-skeletal or skeletal lesions were found. Although the patient underwent surgical resection at the local hospital, the inability to obtain definitive histopathological results—owing to limited local medical resources and geographical constraints-precluded the administration of adjuvant therapy. Unfortunately, recurrence was observed just three months postoperatively. Subsequently, in our institution, the definitive diagnosis of primary breast ES was established through immunohistochemical analysis and fluorescence *in situ* hybridization (FISH). Despite receiving standard chemotherapy and radiotherapy for ES, the patient experienced repeated local recurrences and widespread bone metastases 15 months after her initial diagnosis, ultimately passing away 18 months post-diagnosis.

**Conclusion:**

Primary breast ES represents a rare and aggressive malignancy in children. Early discovery, diagnosis, and treatment are crucial for improving survival rates and life quality for these patients.US, CT, and MRI can facilitate clinical diagnosis and preoperative evaluation. This case highlights the necessity of enhancing clinicians and radiologists awareness about this uncommon condition, especially in pediatric patients and in under-served regions.

## Introduction

In 1921, Ewing first described Ewing sarcoma (ES) as a small round cell tumor originating from endothelial cells. ES is the prototypical and most commonly encountered type within the group of undifferentiated small round cell sarcoma (USRCS). Historically, chest wall tumors were known as “Askin tumors”.Those exhibiting neuronal differentiation were classified as “(peripheral) primitive neuroectodermal tumor (pPNET)”, and those occurring outside the skeletal system were termed EES (1). Prior to 2013, ESFT included classic ES, EES, PNET, Askin tumors, and Ewing-like sarcomas. However, the newer 2013 World Health Organization (WHO) classification updated this by no longer equating PNET with ES (2). Ewing-like sarcomas were also removed from the classification due to their distinct fusion gene and different clinical and pathological characteristics. The most recent WHO Classification of Soft Tissue and Bone Tumors recognizes four categories within this group: ES, round cell sarcoma with EWSR1-non-ETS fusions, CIC rearranged sarcoma, and sarcoma with BCOR alterations (3). At present, ESFTs are commonly divided into classic ES, which affects the bone, and EES, which encompasses PNET and chest wall Askin tumors.

EES was first described by Angervall and Enzinger in 1975 (4). It remains relatively rare, comprising about 15% to 20% of all ESFT cases, resulting in about one case per million in the United States. Notably, EES predominantly affects White individuals, followed by Asians/Pacific Islanders; it is uncommon in the Black population (5). EES can manifest in various body parts, including cutaneous, subcutaneous, soft tissue, paraspinal muscles, the retroperitoneum, kidneys, adrenal glands, pancreas, uterus, and gastrointestinal tract (6). However, the breast is an even more rare primary site for EES (7–9). As is well known, carcinomas represent most malignancies involving the breast, with sarcomas accounting for fewer than 1% of all breast malignancies. Diagnosing breast ES is highly challenging, and there is a lack of definitive treatment guidelines.

The prognosis for EES depends on tumor stage, tumor location and size, patient age, and response to chemotherapy (10). Although the multimodal treatment approach for ES has improved the 5-year survival rate for localized tumors to 70% to 80% (11), the outcomes for patients with metastatic ES remains poor, with 5-year overall survival rates ranging from 20% to 35% (12). Even in primary nonmetastatic cases, 30%–40% of patients experience recurrence, either local, distant, or combined, during follow-up. Survival after recurrence is poor, with 5-year post-relapse survival varying from 15% to 25% (13).

Our case report aims to enhance clinicians' awareness of this type of tumor. Despite its rarity, the likelihood of ES should be considered when evaluating a palpable mass in young females, especially when cytopathological findings indicate the presence of cells of non-breast origin.

## Case report

**Figure 1:**
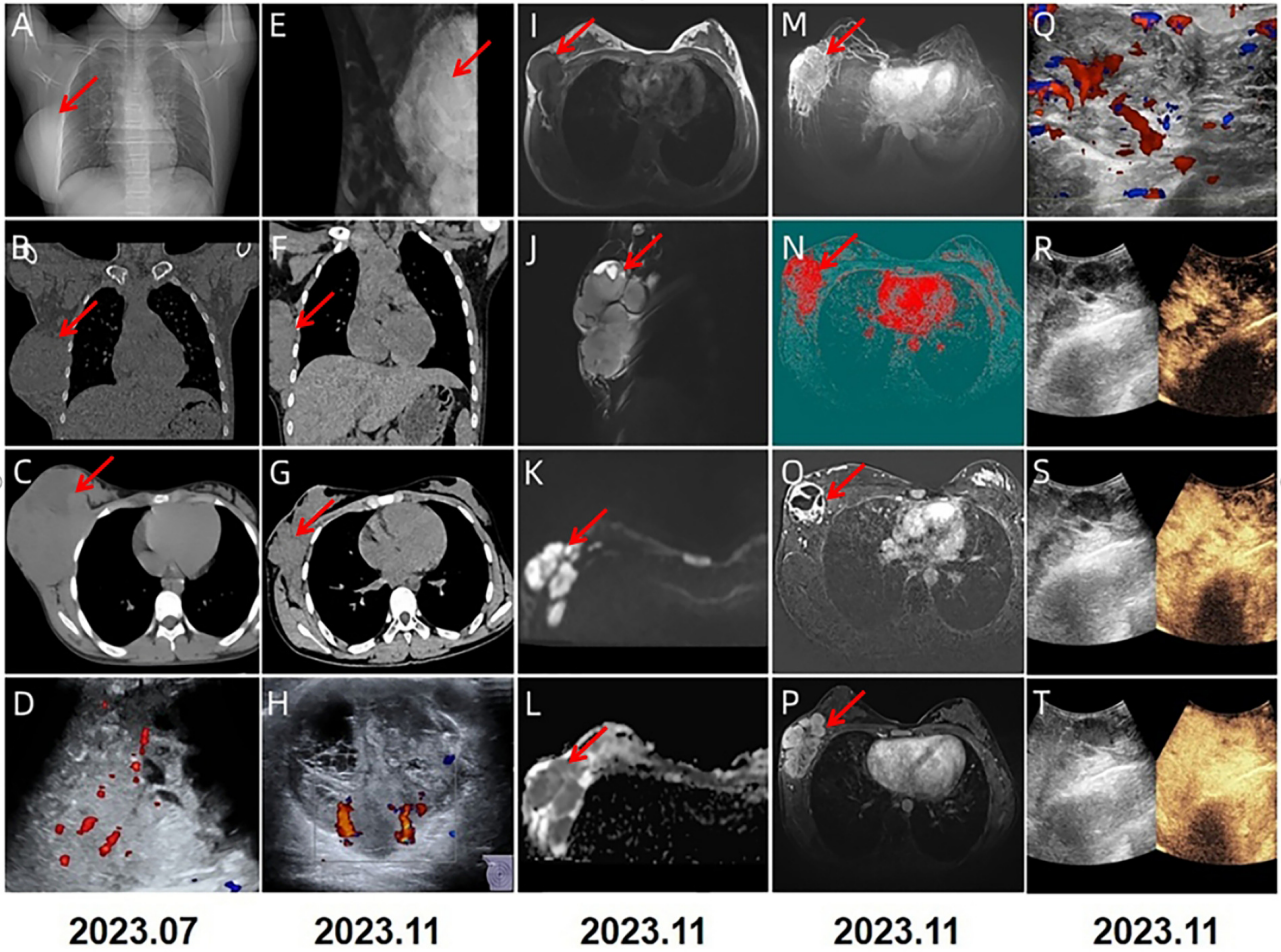
2023.7 **(A–D)**: CT and US showed a solid cystic mass with rich blood supply, measuring 11.8 × 10.3 × 8.5 cm. 2023.11 **(E–T)** multimodal imaging studies were performed on the breast. MG in the medio-lateral oblique **(E)** view identified a lobulated dense mass in the upper inner quadrant of the right breast. CT **(F,G)** scans showed multiple lobulated nodules and masses in the right axilla, breast, chest wall and abdominal wall, with the largest lesion measuring 9.6 × 6.7 × 4.6 cm in size. US **(H)** imaging showed a large, multilobulated hypoechoic to anechoic solid—cystic lesion with rich blood supply. On MRI, these lesions appeared hypointense on T1-weighted **(I)** and hyperintense on fat-saturated T2-weighted sequences **(J)**. They showed diffusion restriction on diffusion-weighted imaging and were dark on apparent diffusion coefficient maps **(K,L)**. Maximum intensity projection **(M)** and colored maps **(N)** depicted the lesions. The tumors showed heterogeneous enhancement with scattered areas of necrosis **(O,P)**. Contrast-enhanced US imaging further revealed the lesions demonstrated progressive enhancement **(Q-T)**.

**Figure 2:**
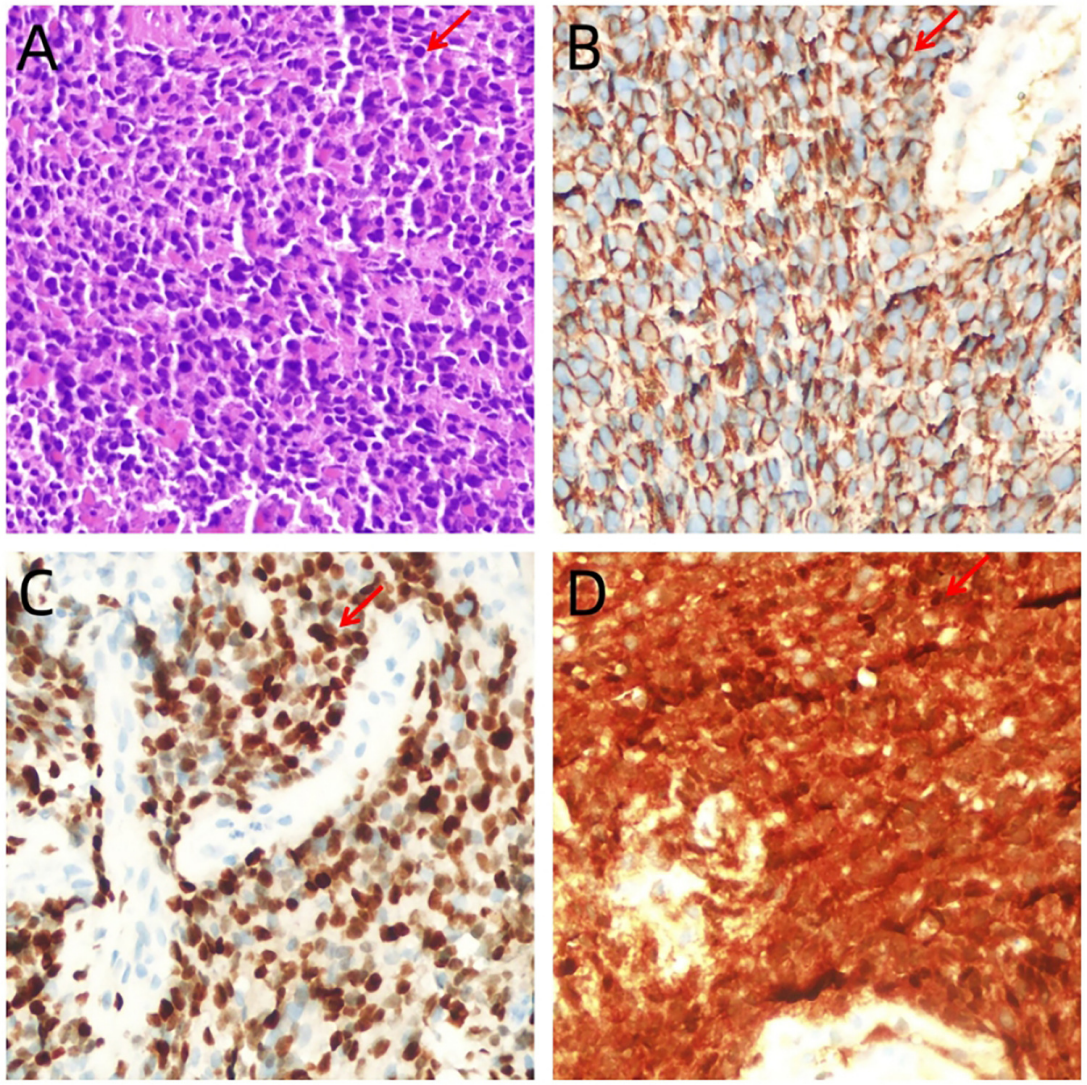
**(A)** Tumor is composed of small, round cells with inconspicuous nucleoli and scanty cytoplasm, which are arranged in sheets or solid nests (hematoxylin-eosin staining, ×400). **(B–D)** Immunohistochemistry ×400: intense and diffuse immunostaining of tumor cells with CD99 **(B)**, NKX2.2 **(C)** and CyclinD1 **(D)**.

**Figure 3:**
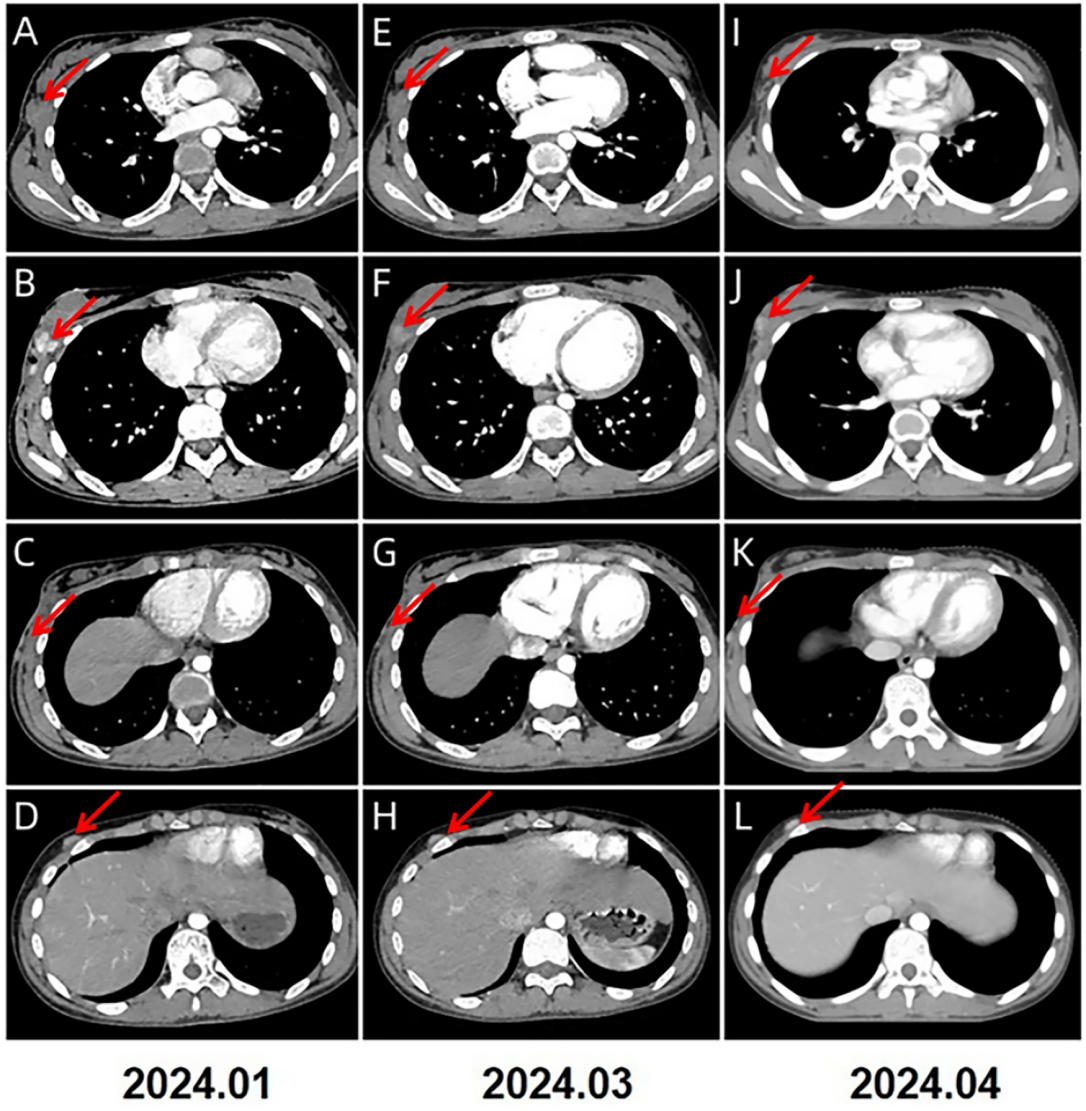
2024.1–2024.4 **(A–L)** CT imaging showed a significant reduction of the lesions in size, with the largest now measuring 1.2 × 1.1 × 1.0 cm (indicated by the red arrows).

A 13-year-old Chinese female adolescent self-detected a slowly growing, palpable lump in the outer quadrant of her right breast, which had been presented for 6 months. She presented in July 2023 to the Breast Unit of Meigu (Southwestern China). Her family history did not reveal any malignancies, especially no history of any breast cancer or rare tumors. On physical examination (PE), a lump was observed in the outer quadrant of her right breast. It was mobile, soft, non-tender, with normal areola and nipple. The mass was 15 × 15 cm in size. There was no axillary or cervical lymphadenopathy, and the rest of the clinical examination was unremarkable. Blood testing revealed a marked rise in lactate dehydrogenase (LDH) to 437 U/L (upper limit of norm <240 U/L), and the alkaline phosphatase (ALP) level was elevated to 220 U/L (upper limit of norm <141 U/L). Chest CT scans (Figure 1A–C) and bilateral breast ultrasounds (Figure 1D) revealed an 11.8 × 10.3 × 8.5 cm solid cystic mass with high vascularity and absence of calcification. The lesion involved the right pectoralis major muscle without causing bone destruction and was classified as a Breast Imaging Reporting and Data System (BIRADS) 4B lesion. Initially, the lump was suspected to be a fibroadenoma or a phyllodes tumor. During the operation, the lump was found to be adherent to pectoralis major muscle. She underwent a lumpectomy of the right breast with negative margins.The lumpectomy specimen revealed a relatively well-circumscribed tumor, measuring 16 × 13 × 8 cm in size. The tumor's cut surface was grayish tan, fish flesh-like, and slightly friable. Due to limited technical conditions, the pathological specimen was sent to Leshan People's Hospital for further analysis. Hematoxylin and eosin (H&E) staining revealed a malignant tumor with areas of necrosis, and immunohistochemical (IHC) analysis was not feasible due to tissue autolysis. She did not undergo any additional adjuvant treatment following the surgery. By October 2023, she presented with a new lump, accompanied by pain near the surgical scar, and was admitted to our hospital for further care. PE revealed several hard masses around the scar on the right breast. Breast cancer-related tumor markers, including CEA, CA125, and CA153, were within normal limits. Blood serum tests showed a slightly elevated LDH level at 280 U/L and an increased ALP level at 224 U/L. Mammography(MG) identified a large, lobulated, dense mass in the upper-outer quadrant of the right breast, with no microcalcification (Figure 1E). CT and conventional US showed multiple solid cystic masses in the right chest wall with a rich blood supply (Figures 1F–H). Breast MRI revealed multilobulated nodules and masses in the right axilla, breast, chest wall, and abdominal wall, with the largest measuring 9.6 × 6.7 × 4.6 cm. These tumors presented as iso-to hypointense on T1-weighted imaging (T1WI) and inhomogeneously iso-to hyperintense on T2-weighted imaging (T2WI). They showed marked diffusion restriction on diffusion-weighted imaging (DWI). Contrast-enhanced MRI revealed heterogeneously enhancing tumors, with enlargement of the right internal mammary artery and involvement of the pectoralis major muscle. (Figures 1I–P). Contrast-enhanced US imaging indicated that the lesions had a rich blood supply (Figures 1Q–T). US-guided biopsy identified a tumor composed of malignant small round blue cells clustered around blood vessels in a sheet-like pattern with areas of necrosis. IHC staining showed positivity for CD99, NKX2.2, CyclinD1, BRG1, INI-1, and FLI-1, and negativity for EMA, Desmin, S-100, ERG, NKX3.1, PCK, CD3, CD10, CD20, CD34, TdT, Myoglobin and Myogenin. The proliferation marker Ki-67 staining was high at 80% (Figures 2A–D). *EWSR 1* translocation was confirmed using fluorescence *in situ* hybridization (FISH). The tumor's morphological characteristics and IHC profile suggested a diagnosis of EES. Staging evaluations, including a whole-body CT scan and ultrasonography at multiple sites, revealed no evidence of lesions in the bones or any other body parts. She commenced the chemotherapy regimen (cyclophosphamide 1.7 g, doxorubicin 30 mg, vincristine 2 mg, day 1, q 21d) and after 4 cycles of chemotherapy, there was more than 80% reduction in the size of the lesions. (Figure 3).The levels of LDH and ALP decreased gradually to 207 U/L and 97 U/L, respectively. However, a few small residual lesions attached to the chest wall remained, and additional treatment, consisting of radiotherapy to the chest wall (PTVI-1 50 Gy/ PTVI-2 60 Gy, 30#, 1.8 Gy/2.0 Gy) was given from May 1st to June 7th. A CT simulation scan conducted on April 10th before radiotherapy (Figures 3I–L) revealed that the lesions were stable. However, a radiotherapy planning CT scan conducted on April 29th showed that the lesions had increased again. The levels of LDH and ALP increased again, to 258 U/L and 175 U/L, respectively. During radiotherapy, the lesion's size remained relatively stable, with an increase in the levels of LDH to 289 U/L and ALP to 219 U/L following treatment and a subsequent decrease upon its completion, to 185 U/L and 179 U/L.

**Figure 4:**
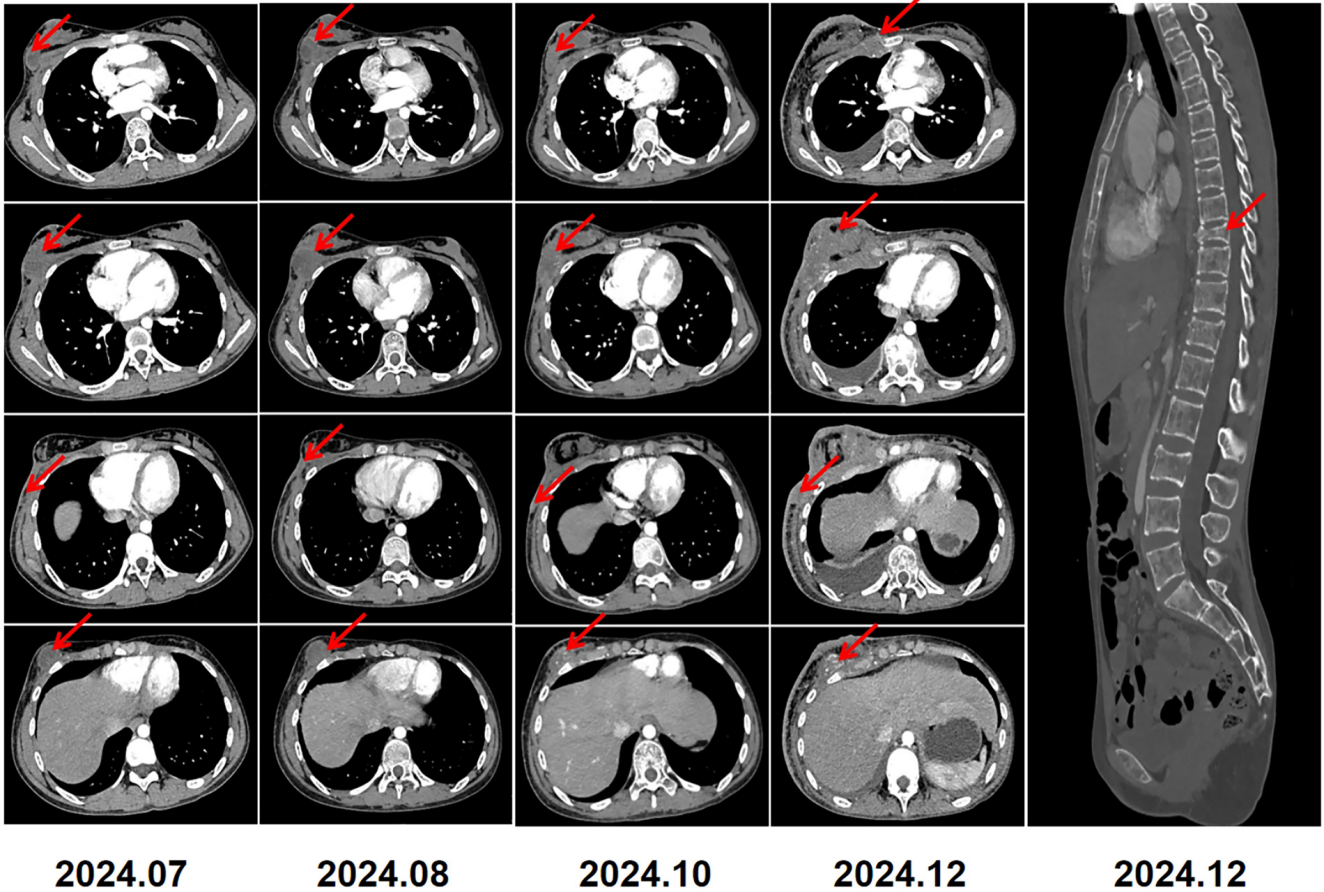
2024.7–2024.12: serial CT imaging demonstrated tumor recurrence involving the right breast, chest wall, and abdominal wall. Diffuse thickening of the overlying right breast skin was noted. Bilateral pleural effusions, small in amount, were present. Imaging also revealed diffuse lytic bone destruction with a pathological compression fracture of the T9 vertebra.

Two months after radiotherapy, follow-up contrast-enhanced CT revealed that the lesions had enlarged compared with the radiation scans, with the largest measuring 3.6 × 3.9 × 4.2 cm in size and having little blood supply (Figure 4 2024/7). The level of LDH increased again to 281 U/L and ALP was stable to 167 U/L. Biopsies at 6 o'clock and 9 o'clock positions in the right breast were performed, revealing the presence of small blue round cells at the edge with 80% areas of necrosis in the center. IHC testing revealed positivity for CD99, NKX2.2, CyclinD1, and FLI-1, and negativity for EMA, Desmin, S-100, NKX3.1, CK-P, SMA,WT-1. The proliferation index Ki-67 was high at 70%. The results confirmed that the lesions had recurred again. Given severe bone marrow suppression in chemotherapy and poor response to radiotherapy, she was recommended for subsequent surgical resection. However, she refused operation and only received anlotinib chemotherapy (12 mg, qd, day 1 to day 14, followed by a 1-week break) for several cycles. A series of follow-up CT scans revealed that the lesions of the right breast,the right chest wall and the right abdominal wall increased significantly (Figure 4 2024/8-2024/12). Additionally, there were diffuse osteolytic bone metastases in the chest and abdomen, along with bilateral pleural effusion. The levels of LDH and ALP increased to 1702 U/L and 264 U/L, respectively. Unfortunately, we learned through a telephone follow-up that this girl passed away 18 months post-diagnosis.

## Discussion

ES/PNET of the breast is challenging to diagnose and treat due to its rarity. This case was unique due to its unusual location, the extremely young age of the patient (13 years), and its rarity in a minority population. In our case, factors such as a rural population with low literacy, a lack of understanding of this disease among clinicians and radiologists, and geographical constraints contributed to the delayed presentation at our hospital. Furthermore,we comprehensively reviewed the related articles in PubMed. Our study aims to provide appropriate education and counseling about this disease, as it is critical for the early diagnosis and treatment of individuals living in rural areas.

To our knowledge, this patient is the 19th documented case of primary ES of the breast in the medical literature.We reviewed data from 19 female patients with primary breast EES/ PNET, with a median age of 36.2 years (range 13–61 years) (Table 1) (7–9, 14–28). Popli et al. (16) reported a case involving a 14-year-old girl with primary ES of the breast. The age at presentation in our case (13 years) was younger than those previously reported. Furthermore, similar to Askin tumors, breast EES/PNET demonstrates a clear female predominance. In our study, there was no right-left predominance in tumor location, with a median diameter of 8.0 cm, which was slightly larger than the sizes previously reported in the literature. Although Majid et al. (29) reported a case of bilateral breast metastatic EES/PNET, bilateral primary EES/PNET of the breast have not been previously reported.

**Table 1:** Case reports of primary breast Ewing sarcoma published in the literature.

Case no	Authors	Published year	Age/gender	Location	Size (mm)	Surgery	Treatment	Follow up
1	Sezer et al. (7)	1999	60/F	Right breast	50	Right mastectomy	Adjuvant chemoradiation	9 months No recurrence
2	Maxwell et al. (8)	2006	35/F	Inner left breast	14	Surgical excision	Adjuvant chemotherapy	30 months No recurrence
3	Tamura et al. (9)	2007	47/F	In the upper inner to outer quadrants of left breast	21	A total mastectomy with axillary lymph adenectomy (Bt + Ax)	Adjuvant chemoradiation	6 months No recurrence
4	da Silva et al. (14)	2008	35/F	Upper left breast quadrant	120	Inoperable	Adjuvant chemoradiation	Local recurrence and death
5	Ko et al. (15)	2009	33/F	Upper inner quadrant of the left breast	25	Surgical excision	NA	6 months No recurrence
6	Popli et al. (16)	2009	14/F	Upper outer quadrant of right breast	120	Surgical excision	NA	NA
7	Vindal et al. (17)	2010	26/F	Medial quadrant of right breast	22	Surgical excision	Adjuvant chemoradiation	36 months No recurrence
8	Machado et al. (18)	2011	50/F	Subcutaneous breast	25	Surgical excision	None	NA
9	Chuthapisith et al. (19)	2012	46/F	Upper right breast	40	Radical mastectomy	Adjuvant chemoradiation	Lung metastases and death
10	Sahoo et al. (20)	2013	36/F	Right breast	80	Right breast and pectoralis major resection	Adjuvant chemoradiation	18 months No recurrence
11	Ikhwan et al. (21)	2013	33/F	Left breast	Occupies the whole left breast	Unsurgical	Adjuvant chemotherapy	Death after three cycles of chemotherapy
12	Meddeb et al. (22)	2014	43/F	Upper quadrant of the outer left breast	130	A total mastectomy with axillary lymph adenectomy (Bt + Ax)	Adjuvant chemotherapy	20 months No recurrence
13	Tasli et al. (23)	2014	24/F	Right breast	130	Surgical excision	Adjuvant chemoradiation	Died 8 months after surgery
14	Mahajan et al. (24)	2014	50/F	Right breast	140	Right breast and pectoralis major resection	Adjuvant chemotherapy	8 months No recurrence
15	Changal et al. (25)	2016	35/F	Upper outer quadrant of left breast	40	Localized wide excision of the left breast with axillary lymph node dissection	Adjuvant chemotherapy	6 months No recurrence
16	Srivastava et al. (26)	2016	25/F	Upper inner quadrant of right breast	116	Localized wide excision of the right breast	Adjuvant chemotherapy	50% reduction in lesions after 4 cycles of chemotherapy
17	Ranade et al. (27)	2020	61/F	Upper inner quadrant of left breast	60	Unsurgical	Adjuvant chemoradiation	Died after 2 years
18	Papi et al. (28)	2022	23/F	Upper outer quadrant of left breast	27	Left mastectomy	Adjuvant chemotherapy	3 months No recurrence
19	Our case	2023	13/F	Outer quadrant of the right breast	118	Surgical excision	Adjuvant chemotherapy	11 months Alive with disease

EES/PNET can be evaluated using mammography (MG), ultrasound (US), CT, or MRI. MG and US are commonly used as initial screening tools for breast lumps. In our study as well as in the literature, 14 and 13 patients, underwent MG and US, respectively, and all presented with dense masses without microcalcification or with nonspecific hypoechoic, well-circumscribed lesions with posterior acoustic enhancement. Maxwell et al. (8) described a similar lesion with an apparent hypoechoic tract extending to the skin that was initially misdiagnosed as a benign epidermal inclusion cyst. CT imaging shows a large, non-calcified, soft-tissue mass with a heterogeneous appearance and areas of cystic degeneration and necrosis. On MRI, EES typically appears as low to isointense signal intensity on T1WI and high signal intensity on T2WI, and it exhibits heterogeneous enhancement due to focal areas of hemorrhage or necrosis (30). Our patient displayed similar MRI characteristics; multimodal breast MRI was significantly superior to CT in accurately assessing the extent of the tumor and its blood supply.

The staging workup for EES involves proper imaging of the primary tumor and potential sites of metastasis. Chest CT and ^18^F-fluorodeoxyglucose positron emission tomography (PET-CT) are the most sensitive tools for detecting lung and other distant or nodal metastases. Moreover, PET-CT can be used to monitor tumor response and progression during treatment. MRI is frequently the preferred imaging modality for assessing the primary tumor and local staging. Emission CT (ECT) provides images of the entire skeleton and identifies both benign and malignant bone lesions. Unfortunately, due to our patient's limited financial resources, a PET-CT scan was not performed. However, the patient underwent a whole-body CT scan, which did not affect the patient's treatment or evaluation of treatment efficacy.

Monomorphic small round blue cells characterized by small hyperchromatic nuclei, inconspicuous nucleoli, sparse cytoplasm, and large necrotic regions, define both skeletal ES and EES (31). EES of the breast must be differentiated from neuroendocrine tumors, metaplastic carcinomas, malignant phyllodes tumors, fibroadenomas, and skin adnexal tumors. Our patient was initially misdiagnosed as having a malignant phyllodes tumors or breast cancer; however, IHC testing was not feasible due to technical and geographical constraints.The patient experienced a relapse only 3 months post-surgery, which significantly negatively impacted the prognosis. Based on the experience of this case, it is crucial to establish an early and accurate diagnosis of primary breast ES, which requires a range of techniques, including IHC testing, genetic analysis, and other procedures. Tumor biopsy should be performed at centers equipped with the facilities to provide IHC, solid tumor cytogenetic, and other molecular diagnostic technologies.

Due to the rarity of breast ES, there are no standardized treatment protocols specifically for primary EES/PNET of the breast. A multiple modalities, including surgery, chemotherapy, and radiation therapy, is considered the most appropriate treatment approach (32). Wide excision to achieve negative margins is the standard surgical approach. Palliative resection can be performed even in the presence of metastases. Furthermore, Ko et al. (15) and Popli et al. (16) reported favorable outcomes in patients with small, localized tumors that were treated by wide excision of just the affected breast. In contrast, studies by da Silva et al. (14), Ikhwan et al. (21), and Ranade et al. (27) have shown that patients who only underwent biopsy without surgical resection, despite receiving chemotherapy, had fatal outcomes.

Previous reports thought that despite the use of adjuvant treatments, local and pulmonary relapses are common, and the prognosis for primary breast EES/PNET is generally poor (8, 9). The outcome is among the worst of all breast cancer subtypes and is even poorer than that of other sites of EES (27). However, in our study, at the time of diagnosis, most patients were in the early stage of the disease and had no distant metastasis (17/19, 89%). The prognosis of these patients were good. Unfortunately, our patient, due to limited medical and financial resources, did not receive effective radiotherapy and chemotherapy after the initial surgery, and experienced recurrence only three months post-operation. Therefore, early diagnosis and early treatment are very important for the patients with breast ES.

Several risk factors are associated with worse prognosis in EES, including older age, pelvic involvement, elevated white blood cell count, increased LDH, and low hemoglobin levels at the time of diagnosis (33). Notably, in our case, LDH and ALP levels exhibited a consistent positive correlation with tumor burden throughout the disease course. However, this relationship was complex: ALP levels did not demonstrate a proportional correlation with the extent of bone destruction. Specifically, in the pre-bone metastasis phase of the disease, ALP levels were observed to rise in parallel with increasing extraskeletal tumor burden. In contrast, in the advanced stage, despite the presence of widespread osteolytic bone metastases in the chest and abdomen, the ALP level remained relatively modest, increasing only to 264 U/L. This suggests that the dynamic relationship between ALP levels and EES prognosis warrants further investigation.

Our study has several limitations. The patient did not undergo a whole-body PET-CT scan at the time of the patient's first recurrence, despite having a whole-body CT. Secondly, the FISH results of gene fusion for the patients were not obtained.

## Conclusion

Pediatric breast Ewing sarcoma is an extremely rare condition. However, it must be considered in the differential diagnosis of a palpable breast mass in young females, particularly when elevated levels of lactate dehydrogenase (LDH) and alkaline phosphatase (ALP) are present. Clinicians should enhance their awareness of this disease to facilitate early diagnosis and treatment.

## Data Availability

The datasets presented in this study can be found in online repositories. The names of the repository/repositories and accession number(s) can be found in the article/Supplementary Material.
